# Unspecific binding of cRNA probe to plaques in two mouse models for Alzheimer’s disease

**DOI:** 10.1186/s12952-016-0065-9

**Published:** 2016-12-16

**Authors:** Anne Schaarschuch, Christoph Redies, Nicole Hertel, Molecular Anatomy and Dysfunction of Mouse Development Group

**Affiliations:** Institute of Anatomy I, Friedrich Schiller University School of Medicine, Jena University Hospital, 07743 Jena, Germany

**Keywords:** In situ hybridization, Alzheimer’s disease, Cell adhesion, Cadherin, Plaques, Unspecific binding, APP/PS1, APP23

## Abstract

**Background:**

Alzheimer’s disease (AD) is characterized by the pathological deposition of amyloid-β (Aβ) protein-containing plaques. Microglia and astrocytes are commonly attracted to the plaques by an unknown mechanism that may involve cell adhesion. One cell adhesion family of proteins, the cadherins, are widely expressed in the central nervous system. Therefore, our study was designed to map the expression of cadherins in AD mouse brains. A particular focus was on plaques because diverse mRNA-species were found in plaques and their surrounding area in brains of AD patients.

**Methods:**

In this study, we used in situ hybridization to visualize cadherin expression in brains of two mouse models for AD (APP/PS1 and APP23).

**Results:**

A variable number of plaques was detected in transgenic brain sections, depending on the probe used. Our first impression was that the cadherin probes visualized specific mRNA expression in plaques and that endogenous staining was unaffected. However, control experiments revealed unspecific binding with *sense* probes. Further experiments with variations in probe length, probe sequence, molecular tag and experimental procedure lead us to conclude that cRNA probes bind generally and in an unspecific manner to plaques.

**Conclusions:**

We demonstrate unspecific binding of cRNA probes to plaques in two mouse models for AD. The widespread and general staining of the plaques prevented us from studying endogenous expression of cadherins in transgenic brain by in situ hybridization.

**Electronic supplementary material:**

The online version of this article (doi:10.1186/s12952-016-0065-9) contains supplementary material, which is available to authorized users.

## Background

With more than 25 million people affected worldwide, AD is one of the most common age-dependent neurodegenerative disorders [1]. Its pathological hallmarks are plaques and neurofibrillary tangles in the brain. Plaques represent protein accumulations, which mostly contain Aβ peptides. Commonly, plaques are surrounded by dying neurons and neuroglia, like microglia and astrocytes [2–4]. The mechanism, by which microglia are attracted by aggregated Aβ and attach to the plaques, is unknown. One possibility is that cell adhesion molecules play a crucial role. Cadherins, a family of calcium-depended cell adhesion proteins, are widely expressed in the central nervous system. They play an essential role in the formation and organization of the nervous system [5–7]. Studies on post-mortem brains of AD patients revealed that not only the protein, but also the mRNA of the Aβ precursor protein (APP) is present in senile plaques [8]. Ginsberg and colleagues [9, 10] provided evidence for diverse mRNA species in plaques and in their surrounding area by the use of in situ hybridizations. In further studies, it was shown that the mRNA for APP was present in the diffuse and immature state of the plaque and that its mRNA regulation is altered [11].

The aim of the present study was to investigate the expression of multiple types of cadherins and to detect possible alterations in brains from mouse models of AD. We used the in situ hybridization technique to map mRNA expression in the brain and other organs. With this very popular and highly useful method, we obtained plaque staining of different intensities, depending on the individual probe used. The initial conclusion drawn from this differential staining pattern was that the cadherin probes bind to plaques in a specific manner. However, careful control experiments, including the use of *sense* probes, demonstrated that the plaque staining was unspecific. Additional controls with probes that varied in length, sequence and the molecular tag, confirmed the unspecific character of the RNA probe binding to the plaques.

## Methods

### Animals

All procedures were performed in accordance with institutional guidelines and national laws on the treatment of animals in research. We used the minimum number of animals necessary to produce reliable scientific data.

For the present study, 6 hemizygous APP/PS1 mice (all female) and 4 wild-type littermates (2 female and 2 male) were used. Differences in the expression patterns between the genders were not detected. In the present study, we show results for advanced amyloidosis at 6 months of age and for a severe state at 12 months of age only. Data for 3-months and 9-months old mice are not provided in the present work (unpublished data) in order to avoid repetitions of similar results. In addition, brains from 18.5-months old APP23 mutant mice (one wild-type and two hemizygous animals; all female) were used. Both mouse lines (kindly provided by Dr. Christoph Kaether, Leibniz Institute of Age Research/Fritz Lipmann Institute, Jena, Germany) have a C57BL/6 background and were originally generated by the groups of Dr. Mathias Jucker and Dr. Matthias Staufenbiel [12;13]. In the APP/PS1 line, human KM670/671NL-mutated APP (= Swedish double mutation) and L166P-mutated PS1 are coexpressed under the murine Thy-1 promotor. In this mutation, amyloid deposits can be detected from 2–4 months of age onward [12]. Mice of the APP23 line express the same Swedish mutated APP under a murine Thy-1 promoter and plaque deposition starts to develop at 6–8 months of age [13].

### Cloning of cDNA fragments of Pcdh8

Template RNA was isolated from the brain of a male C57/BL6J adult mouse by using TRIzol reagent according to the manufacturer’s instructions (Invitrogen, Darmstadt, Germany). Obtained RNA concentration was measured by a spectrophotometer.

Primer pairs for shortened Pcdh8 probes were designed by using the Lasergene® Genomics Suite Software (DNASTAR®, Madison, WI). In order to obtain fragment 1 (F1), which contains the first 812 bp of the open reading frame, the following primers were used: Forward: ATT TAG TCT CTG CTG GGT GCT CTC and reverse: GGG CGC CGA AGG TGA AC. Another primer pair (forward: AGG CCC GGG ATG CTG ACG AA, and reverse: GAC GCT CTG CAA CCC TAC TGT) was used to obtain a second fragment (F2), which represents the last 848 bp of the open reading frame. Reverse transcriptase-polymerase chain reaction (RT-PCR) was performed with the ONE Step RT-PCR Kit (Qiagen, Hilden, Germany) with the following parameters: Reverse transcription at 50 °C for 30 minutes, inactivation of the transcriptase at 95 °C for 15 minutes, followed by 30 cycles of amplification (denaturing for 45 seconds at 94 °C, annealing for 45 seconds at 50 °C for F1 and 60.2 °C for F2, and extension for 1.5 minutes at 72 °C). The correct size of the generated fragments was verified by agarose gel electrophoresis. Fragments were eluted by MinElute Extraction Kit (Qiagen) and cloned into a customized pCR^®^II-TOPO vector by using the TOPO TA Cloning Kit (Invitrogen), following the manufacturer’s instructions. Successfully integrated fragments were sequenced by a commercial company (Eurofins MWG Operon, Ebersberg, Germany) by using specific internal primers. Sequences were verified by using the NCBI-BLAST program [14].

### cRNA probe generating

The plasmids listed in Table 1 were used as templates for the in vitro synthesis of cRNA probes that were labelled with either digoxigenin (DIG) or fluorescein (Fluo). Nonradioactive *sense* and *antisense* probes were produced with the DIG RNA Labeling Kit or Fluo RNA Labeling Kit (Roche Diagnostics, Mannheim, Germany), respectively, according to the manufacturer’s instructions. Quick Spin columns (Roche Diagnostics) were used for purification of the probes. Their correct size was verified by agarose gel electrophoresis.

**Table 1:** Plasmids used for generating cRNA probes

Name	Plasmid	Position of sequence	Accession number	Reference
mouse Cdh2	bMN3-KS+ mouse N-Cdh	333–1313	NM_007664.4	[27]
mouse Cdh11	BSSK11	452–2840	D31963	[28]
mouse Cux-2	pBC SK Cux2 5’	737–1895	U45665	[29, 30]
mouse ER81	BSK-mouse er81	668–3168	NM018781	[31]
mouse Pcdh8	TOPOII-mPcdh8	201–1901	NM001042726	[32]
mouse Pcdh10	mOLe10	full length	U88549	[33]
chicken Pcdh10	pBSVSK-ΔN2257	2259–3899	AF334802	[34]

### Staining of sections

APP/PS1 mice were deeply anesthetized with chloroform and decapitated for brain dissection. APP23 mice were anesthetized with an intraperitoneal overdose injection of pentobarbital (300 mg/kg body weight) and the brains were dissected. Collected brains were fresh frozen in 2-methyl butane chilled to about −40 °C on dry ice and stored at −80 °C. For cryosectioning, frozen brains of APP/PS1 and APP23 were embedded in Tissue-Tek® OCT™ compound (Sakura Finetek Germany, Staufen, Germany), cut at 20 μm thickness on a refrigerated microtome and collected on SuperFrost/Plus slide glasses (Menzel, Braunschweig, Germany). To obtain a neuroanatomical overview, thionin staining was carried out on adjacent sections within each brain series, as described previously [15].

### Immunohistochemistry

#### Single-label immunohistochemistry for Aβ

Brain sections were fixed in 4% formaldehyde (FA) diluted in phosphate-buffered saline (PBS) at 4 °C for 30 minutes and subsequently washed with PBS. For blocking of unspecific antibody binding, sections were incubated for 1 hour at room temperature with 2% sheep serum and 1% Triton-X diluted in PBS. Primary antibody α-3552 against Aβ (derived from rabbit serum; a kind gift of Dr. Christian Haass, University of Munich, Munich, Germany) was diluted 1:1000 in blocking solution and applied overnight at 4 °C. Sections were again washed with PBS and incubated with secondary antibody Alexa Fluor® 488 α-rabbit IgG (Invitrogen) diluted 1:1000 in blocking solution, for three hours at room temperature. Final differentiation was performed by PBS washes. Brain sections were mounted in Mowiol solution (Calbiochem-Novabiochem Corporation, La Jolla, USA).

#### Double-label immunohistochemistry for Protocadherin-10 (Pcdh10) and Aβ

All of the mentioned solutions were supplemented with 1 mM CaCl2 and 1 mM MgCl2. Frozen brain sections were thawed for 30 minutes at 37 °C, followed by retrieval in HEPES-buffered salt solution (HBSS, pH 7.4) and immediately put in ice-cold HBSS-buffer. Sections were fixed in 4% PFA/HBSS for 30 minutes at 4 °C and washed twice with Tris-buffered saline (TBS), followed by TBS supplemented with 0.1% Triton X (TBT). Thereafter, slides were incubated in blocking solution (3% skim milk; 2% normal goat serum in TBT) for 1 hour at room temperature. Primary antibodies α-Pcdh10 (5G10; derived from rat serum; a kind gift of Dr. Shinshi Hirano, Center for Developmental Biology (CDB), RIKEN, Kobe, Japan.), diluted 1:300, and α-3552, diluted 1:1000, were applied in blocking solution overnight at 4 °C, followed by washing steps of TBS and TBT. As secondary antibodies, Biotin-SP-conjugated AffiniPure α-rat (dilution 1:300; Jackson ImmunoResearch, 112-065-143) and Goat anti-Rabbit IgG (H + L) Cy5 (dilution:1:1000; Thermo Fisher Scientific, A10523) were used in the same blocking solution and applied for 2 hours at room temperature. Brain slices were then washed again with TBS and TBT and incubated with Alexa Fluor® 488 conjugate-streptavidin (dilution 1:1000; Thermo Fisher Scientific, S-11223) in blocking solution for 2 hours at room temperature. After several washing steps with TBS, sections were counterstained with Hoechst 33342 (Thermo Fisher Scientific) to visualize nuclei and mounted in Mowiol solution.

### In situ hybridization

The in situ hybridization procedure followed established protocols [15, 16]. Brain sections were fixed in 4% FA/PBS at 4 °C for 30 minutes followed by PBS washes and Proteinase-K digestion (1 μg/ml in 100 mM TRIS pH 8.0, 50 mM ethylenedinitrilotetra acetic acid disodium salt dehydrate [EDTA]; Sigma-Aldrich, Steinheim, Germany) for 5 minutes. After PBS-washing steps, post-fixation for 30 minutes in 4% FA/PBS and washing in DEPC-treated water, slides were treated with 0.25% acetic anhydride/PBS. Subsequently, sections were washed with PBS and then hybridized overnight in a humid chamber with 1 μg/ml cRNA probe in hybridization solution (50% formamide, 10 mM EDTA, 3× saline sodium citrate buffer [SSC], 1× Denhardt’s solution, 10% dextran sulfate, 42 μg/ml yeast tRNA, and 42 μg/ml salmon testis DNA) at 70 °C. However, for the F1 and F2 probes, a hybridization temperature of 67 °C was used. After hybridization, sections were washed with 5× SSC at room temperature, followed by incubation in 5× SSC for 30 minutes at 60 °C. For the F1 and F2 probes, all high temperature washing steps were carried out at 57 °C. Afterwards, sections were incubated in 50% formamide/2× SSC solution at 60 °C for one hour. To remove unbound cRNA, sections were washed with NaCl-TRIS-EDTA buffer and treated with 20 μg/ml RNase A in the same buffer for 30 minutes, followed by another washing step with NaCl-TRIS-EDTA buffer. Subsequently, brain sections were again treated with 50% formamide/2× SSC at 60 °C for 40 minutes and afterwards washed with 2× SSC at 60 °C for 30 minutes. The ensuing washing steps were carried out at room temperature in 0.1× SSC for 30 minutes and PBS. For blocking unspecific binding reactions, brain slices were treated with 2% sheep serum/PBS for one hour at room temperature. Sections were then incubated overnight at 4 °C with alkaline phosphatase-coupled anti-digoxigenin Fab fragments or alkaline phosphatase-coupled anti-fluorescein Fab fragments (Roche Diagnostics) diluted 1:2000 in 1% sheep serum and 0.02% sodium azide in PBS. To reduce background staining, slides were washed with TRIS-buffered saline and incubated in NTM solution (100 mM NaCl, 100 mM TRIS pH 9.5, 50 mM MgCl_2_) for ten minutes. Labeled mRNA was visualized by incubating the sections with the substrates 0.03% nitroblue tetrazolium salt (Fermentas, St. Leon-Rot, Germany) and 0.02% 5-bromo-4-chloro-3-indolyl-phosphate, ρ-toluidine salt (Fermentas) in NTM solution for one to three days at room temperature. The reaction was stopped by washing in H_2_O, followed by a rinse with TE buffer. Staining was differentiated by using ethanol and xylenes. Finally, slides were mounted in Entellan (Merck, Darmstadt, Germany).

### In situ hybridization without formamide

The procedure was the same as the one described above, but we excluded formamide from all washing steps, i.e. sections were treated in the 2× SSC solution only. However, formamide was kept in the hybridization solution.

### In situ hybridization with RNase pretreatment

Brain sections were pretreated with 10 μg/ml RNase A in PBS for 30 minutes at 37 °C, followed by three washes with PBS at 37 °C. RNase A was inactivated by incubation with 0.3 U/μl RiboLock (Fermentas) in PBS for 10 minutes at RT, followed by PBS washes. Subsequently, the in situ hybridization was performed exactly as described above.

### Photomicrograph production

Digital photomicrographs of the brain sections were taken with a light transmission and fluorescence microscope (BX40, Olympus) and a digital camera (DP70, Olympus). Digitized fluorescence signal was converted to grayscale pixel values by a computer. Contrast and brightness of the images were adjusted for optimal display of the staining patterns by using the Photoshop software (CS5, Adobe Systems).

For identification of different brain areas, neuroanatomical nomenclature and abbreviations, an adult mouse brain atlas [17] was consulted.

## Results

### Cadherin in situ hybridization results in differential plaque staining in AD mouse models

**Fig. 1:**
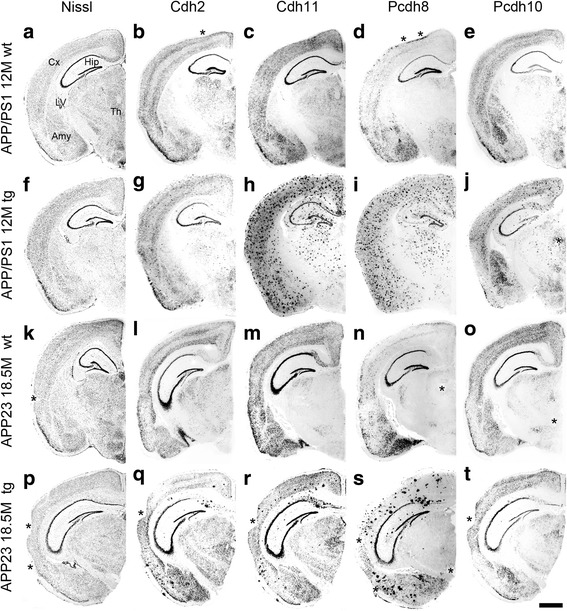
Nissl staining (**a**; **f**; **k**; **p**) and in situ hybridization of (proto-)cadherins in 12-months-old APP/PS1 wild-type mice (**a**-**e**) and transgenic mice (**f**-**j**), as well as in 18.5-months-old APP23 wild-type mice (**k**-**o**) and transgenic mice (**p**-**t**). Nissl staining revealed no differences in cytoarchitecture between the wild-type brains (**a**; **k**) and the transgenic brains (**f**; **p**). Transgenic brains only (**g**-**j**; **q**-**t**) showed staining of plaques by in situ hybridization in both mouse lines, whereas the overall endogenous expression patterns were similar to that of wild-type brains. The asterisks in **b**; **d**; **j**; **k**; **n**; **o**; **p**; **q**; **r**; **s**; **t** indicate artifacts that were induced during dissection of the brains (tissue tears) or collection of the sections on the glass slides (folds and bubbles). Amy, amygdala; Cx, cerebral cortex; Hip, hippocampus; LV, lateral ventricle; tg, transgenic; Th, thalamus; wt, wild-type. Scale bar in t = 1 mm (applies to all panels)

We analyzed the expression patterns of several cadherins with *antisense* cRNA probes. In the present work, we show exemplary staining patterns only for cadherin-2 (Cdh2), Cdh11, protocadherin-8 (Pcdh8) and Pcdh10 in transgenic 12-months-old APP/PS1 mice and wild-type littermates. In Fig. 1, results are compared with 18.5-months-old transgenic and wild-type APP23 mice. The overall staining pattern in wild-type mice was similar in the two mouse strains and did not differ from the endogenous staining patterns described before in wild-type mice [16, 18, 19]. With the cRNA probes of mouse cadherins, we detected a high number of spot-like structures that were exclusively seen in the transgenic brains in both AD mouse lines (Fig. 1g-j; q-t). Because the general distribution of these spots was reminiscent of the distribution of plaques described in AD mouse brains previously [12, 13] and the spot-like staining was never detected in wild-type sections (Fig. 1b-e; l-o), we tentatively identified the spots as plaques. Remarkably, the plaques varied prominently in number and staining intensity between the cadherin probes. While Cdh2 (Fig. 1g; q) and Pcdh10 (Fig. 1 j; t) showed less widespread plaque staining in transgenic AD mouse brains, Cdh11 (Fig. 1h; r) and Pcdh8 (Fig. 1i; s) probes seemed to detect a much greater number of plaques. Nissl staining of adjacent sections (Fig. 1a; f; k; p) revealed no difference in overall cytoarchitecture between the wild-type and transgenic brains or between brains from the two mouse strains. Because there was relatively little difference in the staining patterns between the two lines of AD mouse models, the following results are described for wild-type and transgenic APP/PS1 mice only.

### *Sense* and *antisense* cRNA probes result in similar plaque staining

**Fig. 2:**
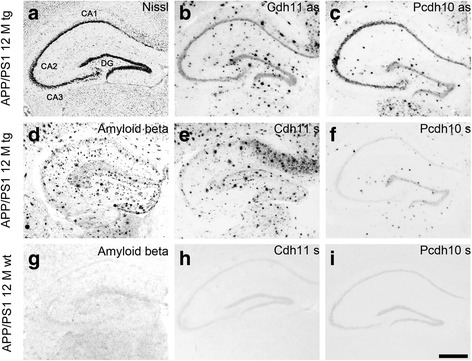
In situ hybridization of transgenic (**a**-**f**) and wild-type APP/PS1 mouse brains (**g**-**i**) at the age of 12 months. Plaque staining is obtained exclusively in transgenic hippocampal formation with *sense* (**e**; **f**; **h**; **i**) and *antisense* (**b**; **c**) probes for Cdh11 and Pcdh10. Amyloid beta immunohistochemistry confirmed the large number of plaques in the transgenic sections (**d**) with no staining visible in wild-type sections (**g**). The Nissl staining in (**a**) indicates a normal hippocampal cytoarchitecture in the transgenic brains. as, *antisense* probe; CA1-3, subdivision 1–3 of the cornu ammonis; DG, dentate gyrus; s, *sense* probe; tg, transgenic; wt, wild-type. Scale bar in i = 1 mm (applies to all panels)

Control experiments with *sense* cRNA probes of the chosen cadherins revealed a high rate of plaque detection in transgenic APP/PS1 mice. This result suggested unspecific plaque staining. To confirm this possibility, adjacent frontal hippocampal sections of transgenic and wild-type APP/PS1 mice were stained with *sense* and *antisense* probes (Fig. 2). A Nissl stain of the hippocampal formation revealed no neuroanatomical abnormalities in transgenic mouse brain (Fig. 2a). Aβ staining of plaques was restricted to transgenic brains (Fig. 2d) and not observed in wild-type sections (Fig. 2g). Both *sense* (Fig. 2e; f) and *antisense* cRNA probes for cadherins (Fig. 2d, c) stained plaque formations in similar number and intensity in transgenic brains. For Cdh11, the *antisense* probe (Fig. 2b) resulted in a normal endogenous staining pattern and, in addition, in a visualization of numerous plaques. The same plaque staining was seen with the *sense* probe for Cdh11, but no endogenous staining was observed (Fig. 2e). Similar results were obtained for Pcdh10 (Fig. 2c; f), but overall plaque staining was less intensive. To confirm that the binding of the *sense* probes was not specific, wild-type sections were hybridized with Cdh11 *sense* probes (Fig. 2h) and Pcdh10 *sense* probes (Fig. 2i), which yielded no endogenous signal.

### Smaller cRNA probes for detection of the same cadherin also show unspecific plaque staining

**Fig. 3:**
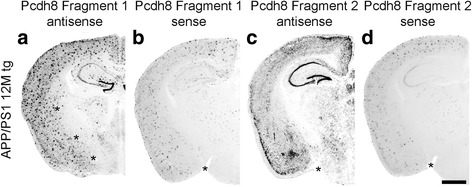
In situ hybridization of Pcdh8 with partial *sense* and *antisense* probes in APP/PS1 transgenic mice at the age of 12 months. Fragment 1 (**a**; **b**) detects the sequence of the first ~ 810 bp from the open reading frame of protocadherin-8, while Fragment 2 (**c**; **d**) detects the last ~ 850 bp. The asterisks in **a**; **b**; **c**; **d** indicate dissection artifacts (see legend to Fig. 1). Scale bar in d = 1 mm (applies to all panels)

The *sense* and *antisense* cadherin probes used for the above in situ hybridization studies were rather long (>1200 bp). To study whether the unspecific plaque binding of the probes depended on probe length or on specific probe regions, we asked whether shorter (partial) cRNA probes for the cadherin sequences resulted in less or even absent unspecific plaque staining. For this purpose, we generated two partial probes from the open reading frame of Pcdh8 and hybridized them with sections from APP/PS1 mice (Fig. 3). Fragment 1 detects the first 812 bp of the open reading frame while fragment 2 hybridizes with the last 848 bp. For both *antisense* probes (Fig. 3a; c), the brain sections revealed the same endogenous staining as the longer Pcdh8 probe of about 1700 bp (Fig. 1i). In addition, the corresponding *sense* probes for fragment 1 (Fig. 3b) and fragment 2 (Fig. 3d) showed plaque staining similar to that described for the longer probes of Cdh11 and Pcdh10 (Fig. 2e: f).

### Unspecific staining behavior is independent of the detected RNA

**Fig. 4:**
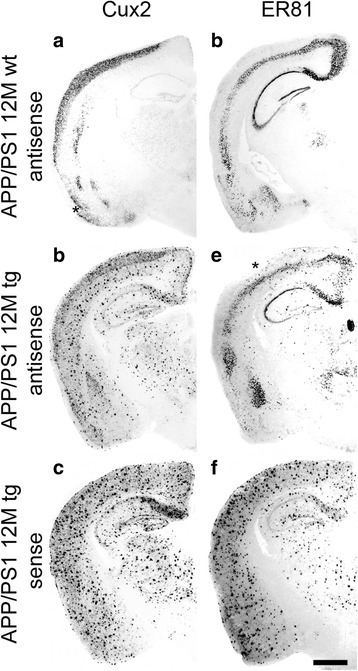
Expression pattern of the transcription factors Cux2 and ER81 in brains of 12-months-old APP/PS1 transgenic (**b**; **c**; **e**; **f**) and wild-type (**a**; **d**) mice. Labelled plaques are seen for both probes with equal intensity for *sense* and *antisense* probes. The asterisks in **a** and **e** indicate dissection artifacts (see legend to Fig. 1). Scale bar in f = 1 mm (applies to all panels)

We next asked whether the unspecific plaque staining is a feature unique to probes detecting cell-adhesion molecules like cadherins. Therefore, APP/PS1 wild-type and transgenic sections were treated with probes for two transcription factors, Cux2 and ER81 (Fig. 4). In wild-type brains, the endogenous expression patterns for Cux2 (Fig. 4a) and ER81 (Fig. 4b) did not differ from the pattern described in the literature [20, 21]. The endogenous staining was also visible on the transgenic sections. In addition, strongly stained dot-like structures were observed when brains were hybridized with *antisense* probes (Fig. 4b; e). A treatment with *sense* probes showed the same plaque staining but without endogenous staining in the brain (Fig. 4c; f).

### Attempts to eliminate the unspecific binding properties

**Fig. 5:**
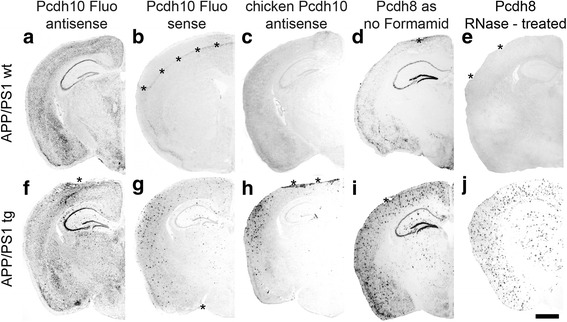
**a**; **b**; **f**; **g**: Results from an attempt to eliminate unspecific plaque binding by using fluorescein-tagged *sense* (**b**; **c**) and *antisense* (**a**; **f**) probes for Pcdh10. **c**; **h**: Results from using *antisense* probes for chicken Pcdh10. **d**; **i**: *Antisense* Pcdh8 staining without formamide in the washing buffers on transgenic and wild-type sections of 6-months-old APP/PS1 mice. **e**; **j**: RNase pretreatment of brain sections before standard Pcdh8-in situ hybridization on wild-type and transgenic brain section of 6-months-old APP/PS1. The asterisks in **b**; **d**; **e**; **f**; **g**; **h**; **i** indicate dissection artifacts (see legend to Fig. 1). tg, transgenic; wt, wild-type. Scale bar in f = 1 mm (applies to all panels)

Because the unspecific plaque binding partially obscured the endogenous staining pattern that was the focus of our study, we attempted to reduce or eliminate it (Fig. 5).

First, we considered the possibility that the digoxigenin tag mediated the unspecific binding of the probes to the plaques. We therefore generated Pcdh10 probes with a fluorescein tag and hybridized transgenic and wild-type APP/PS1 brain sections using the same hybridization procedure as above. The Pcdh10 expression profile in the wild-type brain turned out to be very similar to the one with the digoxigenin tag (compare Fig. 5a to Fig. 1e). Moreover, the same staining pattern for the two different tags was detected in transgenic brain sections (compare Fig. 5b to Fig. 1j ), where plaques were visualized in addition to the endogenous staining. Again, when using the *sense* probe of Pcdh10-fluorescein, the wild-type section showed no staining (Fig. 5b) while the transgenic sections displayed plaque staining only (Fig. 5g).

Second, we asked whether the unspecific binding depended on the species from which the probe was derived. Therefore, we hybridized transgenic and wild-type sections with an *antisense* Pcdh10 probe from chicken. In the sections from transgenic mouse brain (Fig. 5h), plaques were visualized while no staining was seen in the corresponding wild-type section (Fig. 5c). As expected, endogenous staining was not visualized in mouse brain with the chicken probe. Thus, both mouse and chicken probes bind to plaques in mouse sections.

Third, Pardue et al. [22] described that unspecific binding of radiolabeled oligonucleotides can be eliminated by excluding formamide from the in situ hybridization procedure. We therefore modified the in situ hybridization protocol and omitted formamide in the washing steps. With the *antisense* probe for Pcdh8, weaker but specific staining for Pcdh8 can be detected in wild-type sections of 6-months-old APP/PS1 mice (Fig. 5d). In the transgenic brains of a littermate, the unspecific plaque staining was still strong (Fig. 5i).

Forth, as a final proof that the overall plaque staining is unspecific in our experiments, APP/PS1 transgenic and wild-type brain sections were pretreated with RNase A. This pretreatment completely abolished the visualization of endogenous Pcdh8 expression in wild-type and transgenic sections (Fig. 5e; j), but did not eliminate the binding of the probe to the plaques (Fig. 5j ).

### Alternative experimental procedure to investigate cadherin expression in plaques

In Additional file 1: Figure S1, we show a double label immunohistochemistry of Pcdh10 and Aß on a section from 6 months-old APP/PS1 transgenic mouse brain. Aß (red) staining is widely distributed in plaque formations. Co-expression of Pcdh10 (green) in plaques is seen in brain areas only that express Pcdh10 endogeneously (e.g. cerebral cortex). Here, the mantle of the plaques show diffuse co-expression while the dense core shows staining for Aβ only (see insert in A). In areas that are Pcdh10-negative, co-expression is not detected. Thus, there is no ubiquitous, plaque-specific expression of Pcdh10 protein. Unfortunately, only very few antibodies against several cadherins are commercially available. Therefore, it is not possible to investigate a large subset of the cadherin superfamily by immunohistochemistry.

## Discussion

Here, we describe the unspecific binding behavior of RNA probes to plaques in transgenic brains of two mouse models for AD. Due to the lack of binding specificity, it was not possible to investigate the expression of cadherins in and around the plaques by the use of in situ hybridization. Possible factors that might contribute to the present results are discussed in the following sections.

### Unspecific binding to highly compact cellular structures

Control experiments show that the number of detected plaques was as high for *sense* probes as for *antisense* probes. We assume that this result reflects unspecific binding of the RNA probes. Unspecific binding of probes used for in situ hybridization was initially described by Higgs and Wilson [23] who found that probes got stuck in brain areas of compact cell density, e.g. in piriform cortex and hippocampus. The three-dimensional structure of the plaques, which contain Aβ fibres, degraded neurons and microglia, can possibly result in a similarly high density of cells and/or cellular debris, which might attract RNA probes. Furthermore, it has been found that the expression of APP is regulated by RNA-binding proteins [24]. If these proteins are located in and around the plaques, they might act like adhesion traps for RNA. Such a general RNA binding mechanism could explain why every probe that was used in the present study attached to the plaques.

### Maturation state of the plaque

Another factor that might affect RNA detection by in situ hybridization is the state of plaque maturation. Previously, Marcinkiewicz’ study on the detection of mRNA for APP revealed that prominent hybridization signal was obtained only for plaques at an early (diffused) stage of plaque maturation [11]. The low signal obtained in mature plaques lead the author to believe that there was less amount of APP mRNA in mature plaques. Alternatively, because RNA affinity may depend on the plaque stage, we propose that early-stage plaques with their loose structure might be more prone to bind RNA in general. In the present study, we found differential staining of plaques also in individual brain sections. We assume that this differential staining is due to the presence of both early and mature plaques in the sections.

### Effect of endogenous expression on the staining intensity of plaques

Whereas some probes stained nearly all plaques, other probes tended to detect only a few plaques. This differential staining pattern was similar in both transgenic mouse lines and contributed to our initial impression that the staining patterns were specific for individual cadherins. We offer the following possible explanations for this finding: The reduced endogenous staining might originate from differences in the time-course of the substrate reaction between brain sections treated with different probes. On the one hand, for cadherins that are expressed at high levels in the brain, the specific staining will appear fast during the substrate reaction, while the staining of plaques will emerge more slowly. Thus, substrate reaction will be stopped early when endogenous staining has appeared; plaque staining will be less intense. On the other hand, if endogenous expression is weak, the specific staining will appear more slowly, while the staining of plaques emerge faster, resulting in apparently more intense plaque staining when the substrate reaction is stopped. This possibility could also explain why all *sense* probes tend to detect the plaques: Due to the lack of specific binding, the substrate reaction is stopped only after plaque staining has developed. Alternatively or in addition, the reduced endogenous staining might also originate from the high number of plaques, which may capture probes and therefore reduce the amount of probe available for detection of endogenous mRNA. A third possibility for the variation in plaque staining may be a variation in hybridization efficiency of the different probes. While some probes may tend to have a higher binding affinity to their specific sequences, other probes may show less binding specificity and therefore attach more prominently to the plaques.

### RNA probe length

In general, it is believed that shorter fragments of RNA and DNA increase unspecific binding which may explain why oligonucleotides with a length of ~30 bp adhere strongly to plaques, as shown in a study of brain sections of AD patients [22]. Similar results were obtained with biotinylated *sense* and *antisense* oligonucleotides against Aβ [25]. In our study, the initially used probes had lengths of more than >1200 bp, with the aim to decrease unspecific binding. However, all further experiments with variations in probe length (800─3400 bp) always showed the same unspecific plaques staining. This finding suggests that unspecific binding occurs irrespective of probe length.

### Type of the probe tag

Another possible cause for the binding of cRNA probe to the plaques may be the molecular tag of the probe, which might be prone for binding to plaques. To detect the probe, we used digoxigenin, a steroid with a large three-dimensional structure. Digoxigenin is commonly used as a tag for in situ hybridization and immunohistochemistry. To exclude the possibility that digoxigenin caused the unspecific binding, we generated a Pcdh10 probe with the same sequence but with fluorescein, another commonly used molecular tag. This probe turned out to have the same affinity to plaques as the digoxigenin-labelled probe, although fluorescein has a different three-dimensional structure. An investigation with radiolabeled oligonucleotide probes showed similarly unspecific binding to plaques on post-mortem tissue of AD patients [22]. Therefore, we conclude that the attached tag has no effect on the plaque binding.

### Effect of formamide on the unspecific binding

In the study of Pardue and colleagues [22], several steps were varied in the procedure to reduce unspecific binding behavior. The only successful modification was the elimination of formamide during the posthybridization washing steps. Another in situ hybridization study without formamide was successful in a different mouse model for AD and was confirmed by Aβ counterstaining [26]. We also excluded all formamide in the washing steps but were not able to achieve a similar reduction of unspecific binding. One reason might be that the hybridization solution in our study still contained formamide. Without formamide in the hybridization solution, the in situ hybridization failed in our hands.

## Conclusions

Here we described the phenomenon of unspecific binding of cRNA probes to plaques in transgenic brains of two mouse models for AD. Due to the interference by unspecific probe adhesion, it was not possible to investigate alterations in cadherin expression in the transgenic mouse models for AD. One way to approach the investigation of cadherin expression in transgenic AD-mouse lines is the use of protein detection by immunohistochemistry (Additional file 1: Figure 1).

## Additional file

Additional file 1:
**Figure S1.** Double-label immunohistochemistry for Pcdh10 (green) and Aβ (red) on sections from 6-months old APP/PS1 transgenic brains (A). Negative control for an adjacent section (B) was performed by excluding the primary antibodies from the staining procedure. The asterisks in A and B show dissection artifacts (see legend to Fig. 1). The insert in (A) shows a plaque at a higher magnification. Scale bar in B = 200 μm (applies to A, B). Scale bar in the insert = 100 μm. (TIF 27701 kb)

## Abbreviations

AD: Alzheimer’s disease
Amy: Amygdala
APP: Amyloid-β precursor protein
as: *Antisense*
Aβ: Amyloid-β
CA1-3: Subdivision 1–3 of the cornu ammonis
Cdh: Cadherin
Cx: Cerebral cortex
DG: Dentate gyrus
DIG: Digoxigenin
Fluo: Fluorescein
Hip: Hippocampus
LV: Lateral ventricle
Pcdh: Protocadherin
s: *Sense*
Tg: Transgenic
Th: Thalamus
wt: Wild-type
