# Supplementary figures and images for: Unspecific binding of cRNA probe to plaques in two mouse models for Alzheimer’s disease
Source: J Negat Results Biomed. 2016 Dec 16;15:22. doi: 10.1186/s12952-016-0065-9 (PMC5159973; doi:10.1186/s12952-016-0065-9)

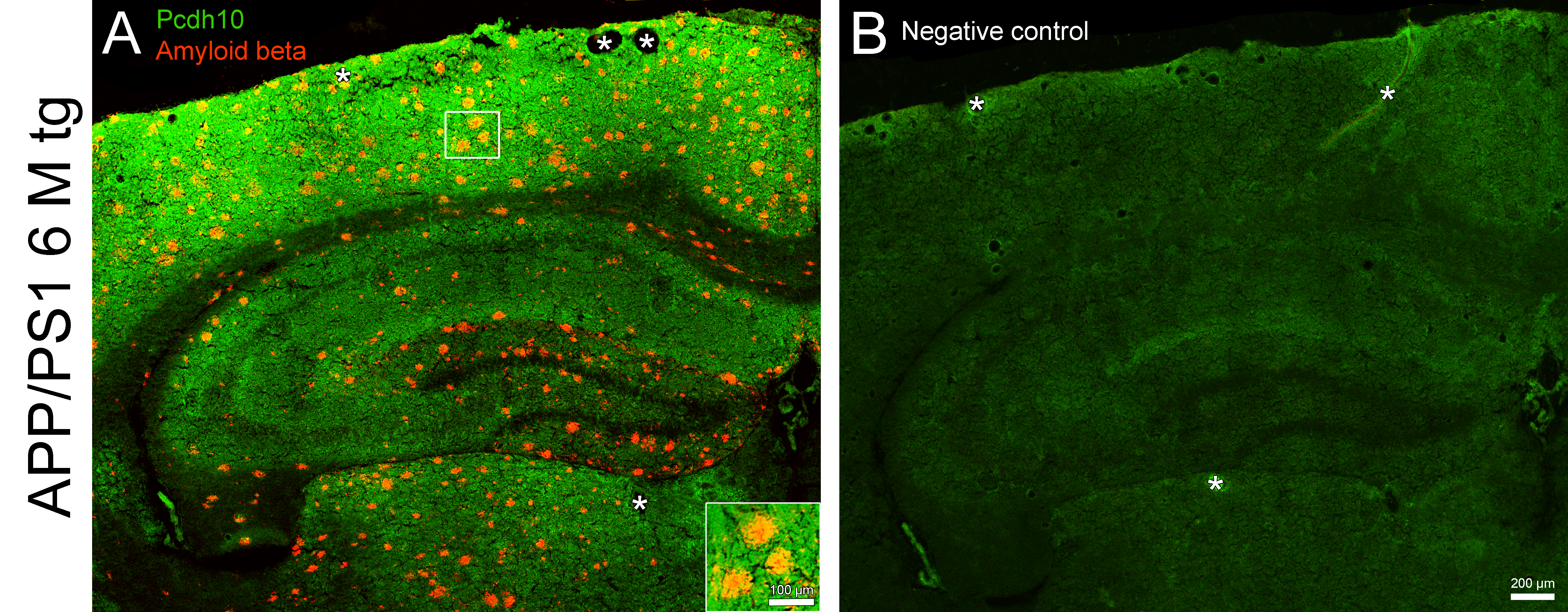

Supplement: Additional file 1: — Figure S1. Double-label immunohistochemistry for Pcdh10 (green) and Aβ (red) on sections from 6-months old APP/PS1 transgenic brains (A). Negative control for an adjacent section (B) was performed by excluding the primary antibodies from the staining procedure. The asterisks in A and B show dissection artifacts (see legend to Fig. 1). The insert in (A) shows a plaque at a higher magnification. Scale bar in B = 200 μm (applies to A, B). Scale bar in the insert = 100 μm. (TIF 27701 kb) [file 12952_2016_65_MOESM1_ESM.tif]
